# CT and MRI features of sarcomatoid urothelial carcinoma of the bladder and its differential diagnosis with conventional urothelial carcinoma

**DOI:** 10.1186/s40644-024-00748-x

**Published:** 2024-08-02

**Authors:** Jiayi Zhuo, Jingjing Han, Lingjie Yang, Yu Wang, Guangzi Shi, Zhuoheng Yan, Lu Yang, Riyu Han, Fengqiong Huang, Xiaohua Ban, Xiaohui Duan

**Affiliations:** 1 Department of Radiology, Sun Yat-sen Memorial Hospital, Sun Yat-sen University, No. 107 Yanjiang Road West, Guangzhou, Guangdong 510120 China; 2 Department of Radiology, Shenshan Medical Center, Sun Yat-sen Memorial Hospital, Sun Yat- sen University, Shanwei, Guangdong 516621 China; 3 Department of Pathology, Sun Yat-sen Memorial Hospital, Sun Yat-sen University, No. 107 Yanjiang Road West, Guangzhou, Guangdong 510120 China; 4 Department of Radiology, State Key Laboratory of Oncology in South China, Guangdong Provincial Clinical Research Center for Cancer, Sun Yat-sen University Cancer Center, 651 Dongfeng Road East, Guangzhou, Guangdong 510060 China

**Keywords:** Bladder tumor, Sarcomatoid urothelial carcinoma, Imaging, Predictive, Diagnosis

## Abstract

**Background:**

Sarcomatoid urothelial carcinoma (SUC) is a rare and highly malignant form of bladder cancer with a poor prognosis. Currently, there is limited information on the imaging features of bladder SUC and reliable indicators for distinguishing it from conventional urothelial carcinoma (CUC). The objective of our study was to identify the unique imaging characteristics of bladder SUC and determine factors that aid in its differential diagnosis.

**Materials and methods:**

This retrospective study enrolled 22 participants with bladder SUC and 61 participants with CUC. The clinical, pathologic, and CT/MRI data from both groups were recorded, and a comparison was conducted using univariate analysis and multinomial logistic regression for distinguishing SUC from CUC.

**Results:**

The majority of SUCs were located in the trigone of the bladder and exhibited large tumor size, irregular shape, low ADC values, Vesical Imaging-Reporting and Data System (VI-RADS) score ≥ 4, the presence of necrosis, and an invasive nature. Univariate analysis revealed significant differences in terms of tumor location, shape, the maximum long-axis diameter (LAD), the short-axis diameter (SAD), ADC-value, VI-RADS scores, necrosis, extravesical extension (EVE), pelvic peritoneal spread (PPS), and hydronephrosis/ureteral effusion (*p* < .001 ~ *p* = .037) between SUCs and CUCs. Multinomial logistic regression found that only SAD (*p* = .014) and necrosis (*p* = .003) emerged as independent predictors for differentiating between SUC and CUC. The model based on these two factors achieved an area under curve (AUC) of 0.849 in ROC curve analysis.

**Conclusion:**

Bladder SUC demonstrates several distinct imaging features, including a high incidence of trigone involvement, large tumor size, and obvious invasiveness accompanied by necrosis. A bladder tumor with a large SAD and evidence of necrosis is more likely to be SUC rather than CUC.

## Introduction

Bladder cancer ranks the eleventh in the global cancer prevalence and is the fourteenth highest cause of death related to cancer [1], which accounts for approximately 90–95% of urothelial carcinoma(UC) [2]. Histologically, UC exhibits histological heterogeneity [3], with 75% being pure UC and the remaining 25% comprising various subtypes [4]. Sarcomatoid urothelial carcinoma (SUC), as one of the rare histopathologic subtypes, represents 0.1–0.3% of all UC of the bladder [4–6]. SUC is characterized by the presence of spindle cells exhibiting high-grade features and differentiation into both epithelial and mesenchymal cell types [3, 7]. It has been reported that SUC is more aggressive than conventional UC (CUC), usually manifesting as an advanced disease entity, thereby resulting in poor prognosis [5, 6]. Clinically, early-stage UC is primarily treated with surgery, while muscle-invasive or advanced UC is commonly managed with platinum-based chemotherapy or neoadjuvant chemotherapy [4–6]. Recently, immunotherapy has shown promising applications in advanced or metastatic UC [8–11], particularly in patients unsuitable for platinum-based therapy [8, 9]. To date, the differences in treatment modalities between SUC and CUC, as well as their response to immunotherapy, remain unclear. However, recent studies suggest that an aggressive multimodal approach may be more effective for SUC, due to its aggressive nature, high malignancy, and poor prognosis [4–6].

Despite advances in understanding the pathological characteristics, therapy, and prognosis of SUCs, there remains a dearth of information on their imaging characteristics due to their rarity [12–15]. Currently, only a recent review referred to the imaging findings of SUC, and showed that both SUC and CUC share overlapping features in imaging and clinical findings [4]. Moreover, due to the rarity of this neoplasm, distinguishing SUC from CUC based on imaging remains unknown. Therefore, it is high desired to depict the imaging features of SUC comprehensively, and to determine the predictive imaging indicators for distinguishing SUC from CUC. In this study, we retrospectively evaluated the radiological findings of SUC and CUC, with a specific focus on identifying CT and MRI features that can differentiate SUC from CUC. The purpose of our study is to improve the understanding of this rare subtype and facilitate its accurate identification through imaging techniques.

## Materials and methods

### Patients

This retrospective study was carried out after the approval of the Institutional Review Board at Sun Yat-Sen Memorial Hospital, and due to its retrospective design, the necessity for obtaining informed consent was exempted. Our datasets included 22 patients diagnosed with bladder SUC (SUC group) over a 10-year period (from May 2013 to May 2022) and 61 patients with bladder CUC (CUC group) over a six-month period (from October 2021 to April 2022). All diagnoses were confirmed through postoperative pathology. To compare their clinical and imaging features, a propensity ratio of approximately 1:3 was used. Exclusion criteria were as follows: patients without bladder MRI or CT scans; imaging examinations were performed for post-treatment surveillance and evaluation; non-bladder origin or primary SUC originating from other sites. The clinical data including age, sex, and symptom of hematuria were collected.

### CT and MR imaging technique

Before their initial treatment, all participants underwent CT and/or MRI examinations with both plain and contrast-enhanced scans. Among the 22 patients with SUCs, 7 underwent CT examination, 14 underwent MRI examination, and 1 patient underwent both MRI and CT examinations. Among the 61 patients with CUCs, 19 had CT examination, 39 had MRI examination, and 3 patients had both MRI and CT scans.

CT examinations were conducted on a 64-slice spiral CT (Sensation 64, Siemens Medical Systems, Germany, *n* = 5) or a 64-slice spiral CT unit (Discovery CT750 HD, GE Medical Systems, USA, *n* = 25). The main parameters included: the tube voltage/current = 120 kV/200 mA, field of view = 300 ~ 350 mm, pitch = 1.20 ~ 1.375, matrix = 512 × 512. After intravenous administration of nonionic contrast material (iopamidol, Jiangsu Hengrui Medicine Co., Ltd., Nanjing, China) at a rate of 3 ml/s with a dosage of 1.2 ml/kg, a contrast-enhanced CT scan was performed with arterial, venous and delayed phases. The acquisition covered the region from the upper pole of both kidneys to the inferior symphysis pubis.

MRI scans were conducted in a 1.5T scanner (Avanto, Siemens Medical Solutions, Germany) for 29 patients, or a 3.0T MR scanner (Skyra, Siemens Medical Solutions, Germany) for 28 patients. For each patient undergoing bladder MRI examinations, axial, sagittal, and coronal fast spin echo (FSE) T2-weighted images (T2WI), axial T1-weighted images (T1WI), and diffusion-weighted images (DWI) were obtained. Following the acquisition of conventional MRI sequences and DWI, Gd-DTPA-BMA (Omniscan, GE Healthcare, Ireland) were intravenously administered (injected dose = 0.1 mmol/kg; injected rate = 2 ml/s), then with a flush of 20 ml saline. Dynamic contrast enhanced MRI (DCE-MRI) sequences were included for 27 patients, which initiated at 30 s post-contrast medium injection, consisting of an additional 6 continuous phases with a temporal resolution of 30 s. Conventional contrast-enhanced T1WI images were acquired after DCE-MRI, and the parameters were the same as those for un-enhanced T1WI. The Table 1 shows a comprehensive overview of the sequences and parameters in bladder MRI.

**Table 1:** Imaging parameters of conventional MRI sequences and DWI, DCE-MRI

	T2WI	T1WI	DWI	DCE-MRI
*Parameter setting at 1.5 T*				
TR/TE (ms)	2800/100	7.03/2.39	4700/75	7.04/1.88
FOV (mm^2^)	350 × 318	380 × 309	280 × 280	260 × 260
Matrix size	384 × 326	320 × 240	160 × 128	224 × 190
Slice thickness (mm)	5.0	3.0	4.0	1.5
Slice gap (mm)	1.5	0.6	0.9	0.3
NSA	3	1	1/5/8	3
B values (s/mm^2^)			0–800–1500	
*Parameter setting at 3.0 T*				
TR/TE (ms)	3200/120	3.98 × 1.29	5400/82	4.03/1.29
FOV (mm^2^)	230 × 230	380 × 285	210 × 210	380 × 285
Matrix size	256 × 256	320 × 240	132 × 132	320 × 240
Slice thickness (mm)	3.5	1.0	4.0	1.0
Slice gap (mm)	0.7	0.2	0.8	0.2
NSA	2	2	1/5/6	2
B values (s/mm^2^)			0–800–1200	

Note—TR, repetition time; TE, echo time; FOV, field of view; NSA, number of signal averaged

### Imaging analysis

The images were independently assessed by two experienced radiologists (B.X. and S.G., with 12 and 10 years of experience, respectively), without access to pathological information. In cases where the two radiologists disagreed, a third radiologist (D.X. with 14 years of experience) was consulted to make the final decision. The following radiologic features of the tumors were analyzed: location, number, shape, size, boundary, necrosis, hemorrhage, calcification, CT-value, signal intensity on T1WI and T2WI, DWI restriction, apparent diffusion coefficient (ADC) value, enhancement degree and pattern, Vesical Imaging-Reporting and Data System (VI-RADS) (1–5), extravesical extension (EVE), invasion of the urethral orifice, ureteral invasion, pelvic peritoneal spread (PPS), hydronephrosis/ureteral effusion and suspicious pelvic lymph nodes. For statistical analysis, tumor location was classified as trigone or nontrigone. The number of tumors included single or multiple. Tumor size was measured as the maximum diameter along its longest axis (LAD) in any direction, the short-axis diameter (SAD) perpendicular to the LAD, and the ratio of SAD to LAD (S/L) [16]. Tumor shape included endophytic, exophytic, flat or mixed [17]. The margin was categorized as well-defined or ill-defined. The degrees of enhancement were categorized as marked, moderate, or mild, and enhancement patterns comprised homogeneous or heterogeneous. Mild enhancement was defined as exhibiting a level of enhancement that was equal to or lower than that of the surrounding muscle or a ∆CT value (calculated as the CT value in the venous phase minus the CT value in the un-enhanced phase) of the lesion less than 30 HU. Moderate enhancement was greater than that of muscle but less than that of adjacent colorectal mucosa on MRI, or a ∆CT value of the lesion ranging from 30 to 60 HU. Marked enhancement was defined as having a resemblance to the enhancement of colorectal mucosa on MRI, or a ∆CT value of the lesion exceeding 60 HU. The CT-value and ADC were determined by delineating an ROI within the tumor as large as possible (excluding cystic degeneration and necrosis). Intratumor calcification was visualized via CT while hemorrhage and necrosis were determined through CT or MRI. The VI-RADS was used in our study to assign scores ranging from 1 to 5, in accordance with established guidelines [17, 18]. For patients without dynamic contrast-enhanced MRI (DCE-MRI) examinations, we still adaptively employed the VI-RADS for assessment (30/57) [19]. A VI-RADS score of ≥ 4 indicates a suspicion for muscle-invasive disease, while a score of 5 suggests the presence of extravesical extension (EVE). Pelvic peritoneal spread (PPS) is distinguished by the existence of enhanced soft tissue that extends along the fascial planes within the pelvic region [16]. The suspicion of lymphatic metastasis arises when the short-axis diameter of pelvic lymph nodes exceeds 1 cm or greater than 0.6 cm with a circular or irregular shape [16].

### Statistical analysis

The SPSS software (version 25.0, Chicago, IL, USA) was utilized for statistical analysis. According to the receiver operating characteristic (ROC) curve analysis, the optimum cutoff value for age was identified as 59.5 years. In terms of pathological data, the presence or absence of muscle invasion was selected for statistical analysis. Clinical and radiologic parameters included age (≥ 59.5 or < 59.5 years), sex (male or female), number (single or multiple), tumor location (trigone or nontrigone), size (LAD, SAD, and S/L), margin (well-defined or ill-defined), shape (endophytic, exophytic, flat, or mixed), CT-value, signal intensity on T1WI/T2WI (isointense, hyperintense, or mixed), ADC-value, pattern (homogeneous/heterogeneous) and degree (mild/ moderate/marked) of contrast enhancement, VI-RADS (1–5), and the presence or absence of hematuria, necrosis, hemorrhage, calcification, DWI restriction, EVE, PPS, invasion of the urethral orifice, hydronephrosis/Ureteral effusion, and suspicious pelvic lymph nodes. Comparisons of the clinical and CT/MRI features between the two groups were conducted via univariate analysis. For continuous variables, independent t-tests or nonparametric tests (Mann-Whitney U test) were employed, and Chi-squared tests were used for categorical variables. Subsequently, a stepwise forward (conditional) multinomial logistic regression analysis was conducted to identify the factors that can differentiate between SUCs and CUCs. The performance of each predictor was assessed by calculating the area under the receiver operating characteristic curve (AUC), sensitivity, specificity, accuracy, positive predictive value (PPV), and negative predictive value (NPV). Statistical significance was determined using a *P*-value threshold of less than 0.05. Furthermore, the diagnostic efficacy of the identified predictors was evaluated through ROC curve analysis, providing a comprehensive evaluation of both sensitivity and specificity.

## Results

### Patients

In the SUC patient cohort, 18 males and 4 females were included, aged between 38 and 89 years (mean age = 66.0 years). In the CUC group, 58 males and 3 females, aged from 30 to 93 years (mean age = 64.0 years), were included. The predominant complaint in both groups was hematuria, with a prevalence of 81.8% (18/22) and 77.0% (47/61) in the SUC and CUC groups, respectively. No significant differences were observed in sex, age, and the occurrence of hematuria between the two cohorts. However, a significant difference was found in the presence of muscle invasion based on pathological findings (*p* < .05). The incidence of muscle invasion was significantly higher in the SUC group (90.9%, 20/22) compared to the CUC group (32.8%, 20/61) (Table 2).

**Table 2:** Differences in the clinical and CT/MRI features between SUC and CUC

	CUC(*N* = 61)	SUC(*N* = 22)	*p* ^#^
*Clinical variables*			
Age (years)			0.182
< 59.5	20(32.8)	4(18.2)	
≥ 59.5	41(67.2)	18(81.8)	
Sex			0.141
Male	58(95.1)	18(81.8)	
Female	3(4.9)	4(18.2)	
Hematuria			0.870
No	14(23.0)	4(18.2)	
Yes	47(77.0)	18(81.8)	
Muscle invasion			< 0.001
Muscle-invasive	20(32.8)	20(90.9)	
Non-muscle-invasive	41(67.2)	2(9.1)	
*CT/MRI variable*			
Location			0.009
Trigone	22(36.1)	15(68.2)	
Nontrigone	39(63.9)	7(31.8)	
Number			0.504
Single	31(50.8)	13(59.1)	
Multiple	30(49.2)	9(40.9)	
Shape			< 0.001
Endophytic	3(4.9)	0(0)	
Exophytic	48(78.7)	8(36.4)	
Flat	2(3.3)	1(4.5)	
Mixed	8(13.1)	13(59.1)	
Size (cm) ^*^			
LAD	3.09(0.56–10.15)	4.70(2.03–8.90)	< 0.001 ^a^
SAD	1.84(0.35–6.26)	3.64(1.48–7.53)	< 0.001 ^a^
S/L	0.71(0.20–0.97)	0.73(0.46–0.92)	0.577 ^a^
Margin			0.462
Well-defined	1(1.6)	1(4.5)	
Ill-defined	60(98.4)	21(95.5)	
Necrosis			< 0.001
No	56(91.8)	10(45.5)	
Yes	5(8.2)	12(54.5)	
Hemorrhage			0.091
No	57(93.4)	17(77.3)	
Yes	4(6.6)	5(22.7)	
Calcification	*n* = 22	*n* = 8	> 0.99
No	13(59.1)	5(62.5)	
Yes	9(40.9)	3(37.5)	
CT-value (HU) ^†^	*n* = 22	*n* = 8	0.309 ^b^
	33.04 ± 5.15	35.38 ± 6.28	
Signal intensity on T1WI	*n* = 42	*n* = 15	0.153
Hypointense	1(2.4)	1(6.7)	
Isointense	38(90.5)	11(73.3)	
Mixed	3(7.1)	3(20.0)	
Signal intensity on T2WI	*n* = 42	*n* = 15	0.153
Isointense	38(90.5)	11(73.3)	
Hyperintense	1(2.4)	1(6.7)	
Mixed	3(7.1)	3(20.0)	
DWI restriction	*n* = 42	*n* = 15	NA ^‡^
No	0(0)	0(0)	
Yes	42(100)	15(100)	
ADC-value (10^− 3^ mm^2^/s)^*^	*n* = 42	*n* = 15	< 0.001^a^
	0.964(0.496–1.988)	0.721(0.352–1.034)	
Enhancement degree			0.348
Mild	1(1.6)	2(9.1)	
Moderate/marked	60(98.4)	20(90.9)	
Enhancement pattern			0.479
Homogeneous	8(13.1)	1(4.5)	
Heterogeneous	53(86.9)	21(95.5)	
VI-RADS (1–5)	*n* = 42	*n* = 15	0.006
1	2(4.8)	1(6.7)	
2	9(21.4)	1(6.7)	
3	11(26.2)	0(0)	
4	10(23.8)	2(13.3)	
5	10(23.8)	11(73.3)	
EVE			0.001
No	44(72.1)	7(31.8)	
Yes	17(27.9)	15(68.2)	
PPS			0.037
No	55(90.2)	15(68.2)	
Yes	6(9.8)	7(31.8)	
Invasion of the urethral orifice			0.516
No	55(90.2)	18(81.8)	
Yes	6(9.8)	4(18.2)	
Ureteral invasion			0.461
No	44(72.1)	14(63.6)	
Yes	17(27.9)	8(36.4)	
Hydronephrosis/Ureteral effusion			0.014
No	50(82.0)	12(54.5)	
Yes	11(18.0)	10(45.5)	
Suspicious pelvic lymph nodes			0.187
No	58(95.1)	19(86.4)	
Yes	3(4.9)	3(13.6)	

Note—Unless otherwise specified, data are numbers of patients, with percentages in parentheses

LAD, long-axis diameter; SAD, short-axis diameter, S/L, short to long axis ratio; EVE, extravesical extension; PPS, pelvic peritoneal spread

^*^ Data were expressed as median (minimum-maximum)

^†^ Data was expressed as the mean ± standard deviation (mean ± SD).

^#^ Chi-square test unless otherwise stated

^a^ Mann-Whitney U test; ^b^ independent sample t-test

^‡^ These values could not be calculated due to no disease-negative patients in each group

### Imaging features of SUCs and univariate analysis

**Fig. 1:**
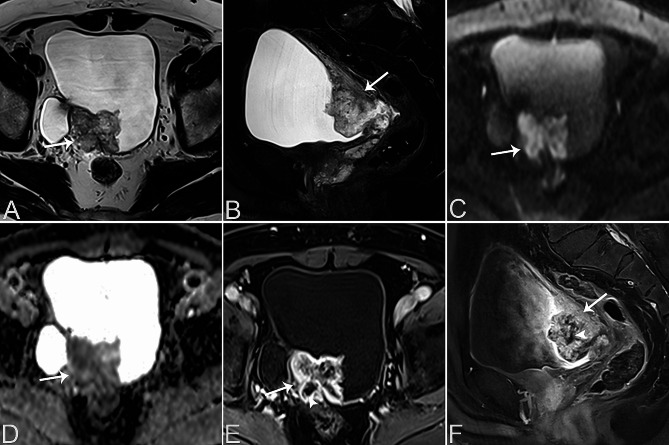
A 70-year-old male patient with bladder SUC in the right posterior wall of the bladder. Axial T2WI (**A**) and sagittal T2WI (**B**) show a lobulated mass in the right posterior wall of the bladder, demonstrating hyperintensity on T2WI (arrows). Axial DWI (**C**) shows the mass with high signal intensity (arrow). Axial ADC map (**D**) shows diffusion restriction of the bladder tumour (arrow). Contrast-enhanced axial (**E**) and sagittal (**F**) T1W images of the bladder tumor (arrows) displays heterogeneous and marked enhancement, with the presence of non-enhancing necrotic areas (arrowheads)

**Fig. 2:**
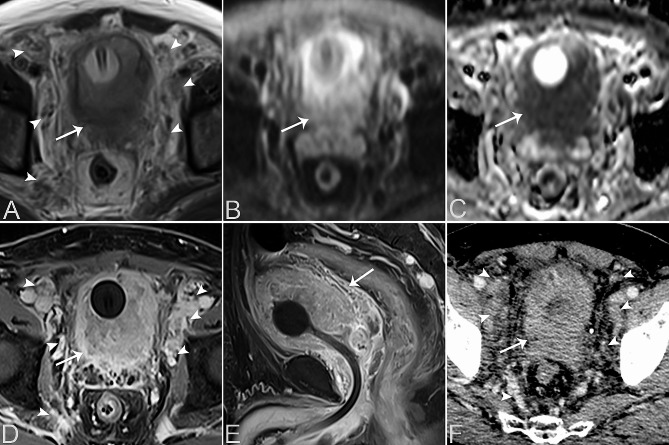
A 63-year-old male patient with bladder SUC involving the entire bladder wall diffusely. Axial T2WI (**A**) shows an ill-defined, irregular mass in the diffuse bladder wall, with the tumor demonstrating iso- or slight hyperintensity on T2WI (arrow). The structure of the bladder wall was blurred with unclear edges. Multiple cord-like and nodular T2 hyperintense lesions were observed around the bilateral pelvic walls and iliac vessels (arrowheads). Axial DWI (**B**) shows the mass with high signal intensity (arrow), and the axial ADC map (**C**) exhibits significant diffusion restriction (arrow). Contrast-enhanced axial (**D**) and sagittal (**E**) T1WI demonstrate obvious heterogeneous and marked enhancement of the mass (arrows), with unclear borders. Axial contrast-enhanced CT image reveals a prominently enhanced tumor in (**F**) the delayed phase. Multiple lymph node metastases were observed around the bilateral pelvic walls and iliac vessels (arrowheads)

**Fig. 3:**
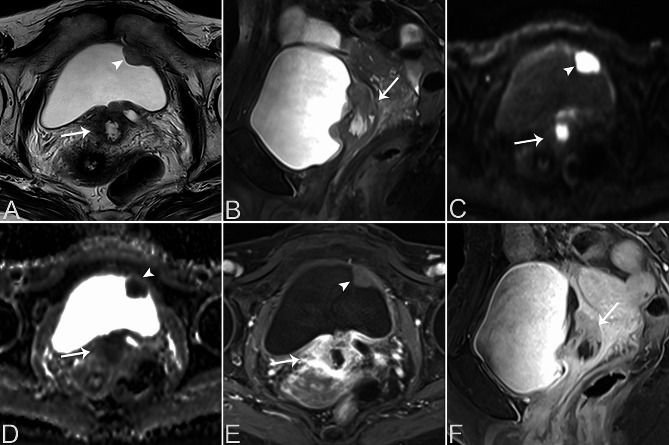
A 61-year-old female patient with bladder SUC in the bladder trigone area and CUC in the left anterior wall of the bladder. Axial T2WI (**A**) and sagittal T2WI (**B**) reveal an exophytic mass in the bladder trigone area, demonstrating heterogeneous slight hyperintensity (arrows) with a central area of higher T2 signal indicative of necrosis. The lesion in the trigone area has unclear borders, involving the lower end of the left ureter with ureteral effusion. The lesion of CUC in the left anterior wall of the bladder appears as a nodular mass with slight hyperintensity on T2WI and clear borders (arrowheads). Axial DWI (**C**) shows both bladder masses with high signal intensity (arrow, arrowhead), and the axial ADC map (**D**) exhibits marked diffusion restriction (arrow, arrowhead). Contrast-enhanced axial (**E**) and sagittal (**F**) T1WI demonstrate heterogeneous and marked enhancement of the trigone area tumor with central necrosis (arrows). The CUC lesion in the left anterior wall of the bladder shows homogeneous mild enhancement (arrowhead)

**Fig. 4:**
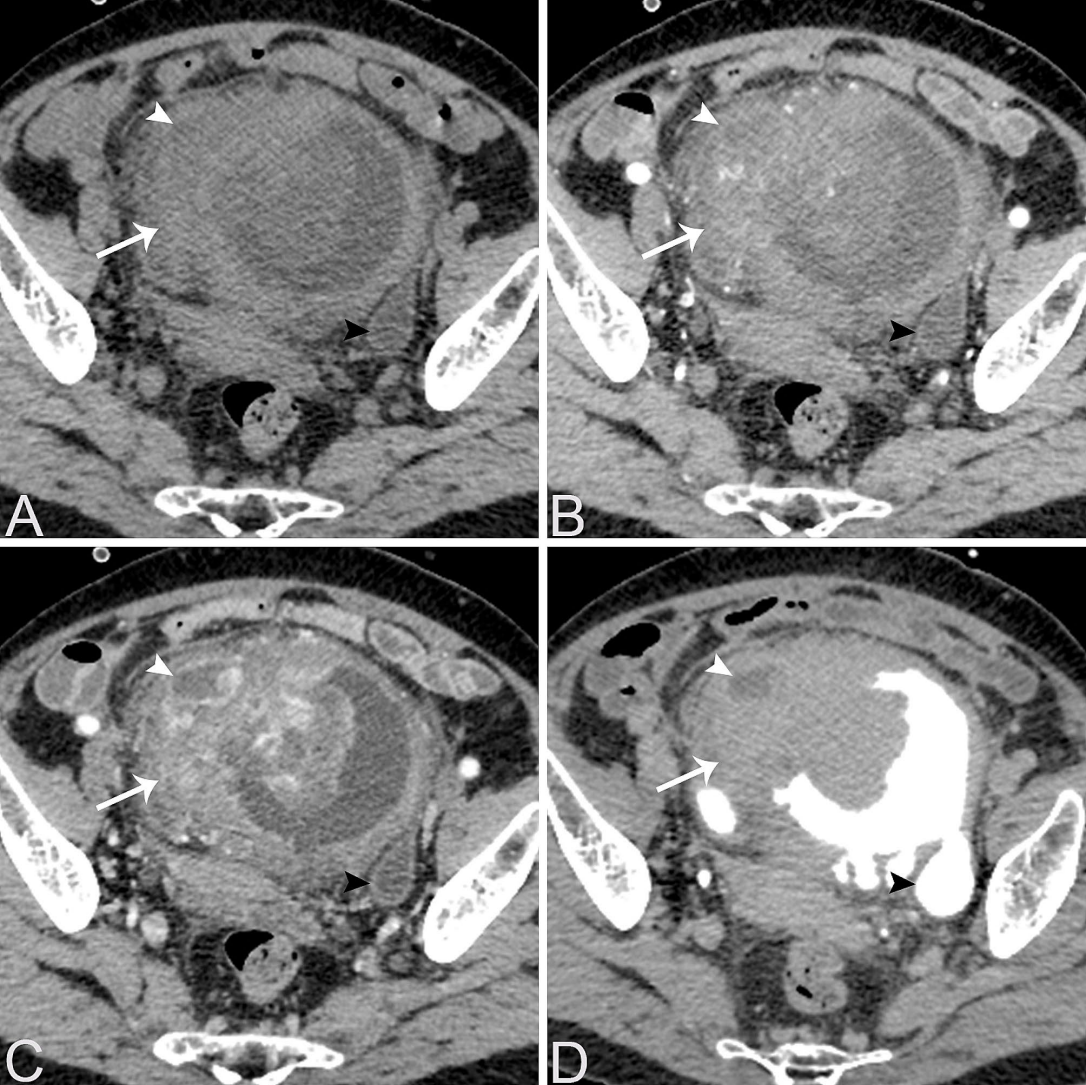
A 69-year-old female patient with bladder SUC involving the right bladder wall diffusely. Axial non-contrast CT (**A**) shows an ill-defined, lobulated, and slightly hypodense lesion in the diffuse bladder wall, predominantly involving the right wall (arrow). Contrast-enhanced CT images in the corticomedullary phase (**B**), nephrographic phase (**C**), and excretory phase (**D**) reveal heterogeneous and marked enhancement of the mass (arrow) with small focal areas of necrosis (white arrowheads). A local bladder diverticulum was noted in the left posterior wall (black arrowheads)

**Fig. 5:**
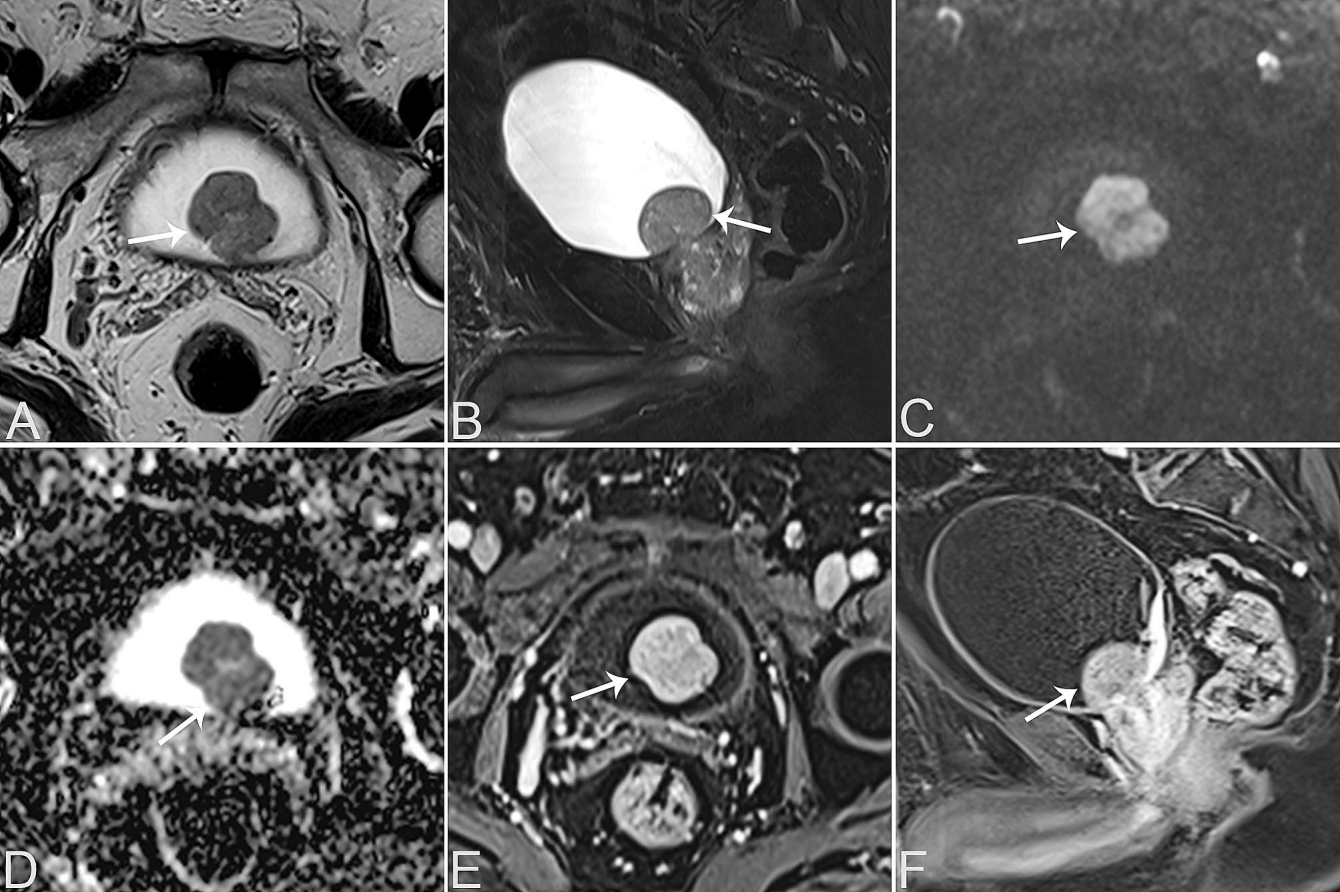
A 74 -year-old male patient with CUC lesion in the bladder neck. Axial T2WI (**A**) and sagittal T2WI (**B**) show a well-defined, oval mass in the bladder neck (arrow). The mass appears slightly hyperintense on T2WI. Axial DWI (**C**) shows the mass with high signal intensity (arrow), and the axial ADC map (**D**) exhibits marked diffusion restriction (arrow). Contrast-enhanced axial (**E**) and sagittal (**F**) T1WI demonstrate the tumor with relatively homogeneous enhancement without apparent necrosis (arrow)

Table 2 provides a summary of the imaging features of both SUCs and CUCs, with representative CT and MRI images are presented in Figs. 1, 2, 3, 4 and 5. Among patients with SUC, 68.2% (15/22) of the lesions were located in the trigone area, while 31.8% (7/22) in nontrigone regions, including 13.6% (3/22) in the lateral walls, 13.6% (3/22) in the posterior wall, and 4.6% (1/22) in the anterior wall. The LAD of SUCs ranged from 2.03 to 8.90 cm, with a median of 4.70 cm, while the SAD ranged from 1.48 to 7.53 cm, with a median of 3.64 cm. Among SUCs, 8/22 (36.4%) patients had exophytic lesions, 1/22 (4.5%) had flat lesions, and 13/22 (59.1%) had mixed lesions. Tumor margin was ill-defined in most of our patients (95.5%, 21/22). Most lesions were solitary (59.1%, 13/22), showing heterogeneous enhancement (95.5%, 21/22), and moderate/marked enhancement (90.9%, 20/22). Necrosis was present in 12 patients (54.5%, 10/22). Among the SUCs evaluated by bladder CT (*n* = 8), calcification was found in 3 patients (37.5%, 3/8). On MRI, SUCs (*n* = 15) showed a non-characteristic signal intensity, as well as the enhancement pattern and degree, but a relatively lower ADC-value (median value = 0.721 × 10^− 3^ mm^2^/s). Additionally, 13/15 (86.7%) of the SUCs were classified as VI-RADS 4 or 5. Hydronephrosis or ureteral effusion was present in 10/22 (45.5%) patients with SUCs, while ureteral invasion was found in 8/22 (36.4%) patients. Among SUCs, 13/22 (59.1%) patients had EVE, 7/22 (31.8%) had PPS, 4/22 (18.2%) had invasion of the urethral orifice, and 3/22 (13.6%) had suspicious pelvic lymph nodes.

The imaging features, including location, shape, LAD, SAD, S/L, ADC value, VI-RADS scores, necrosis, EVE, PPS, and hydronephrosis/ureteral effusion, showed significant differences between the two groups (*p* < .05). Compared with CUCs, SUCs tended to locate in the trigone area with a mixed shape, a larger size, a lower ADC value and necrosis. The lesions of SUCs were preferred to have a higher VI-RADS scores, EVE, PPS, and hydronephrosis/ureteral effusions. All these above imaging findings indicated that SUCs tend to exhibit a more invasive nature.

### Predictive factors for differential diagnosis

**Fig. 6:**
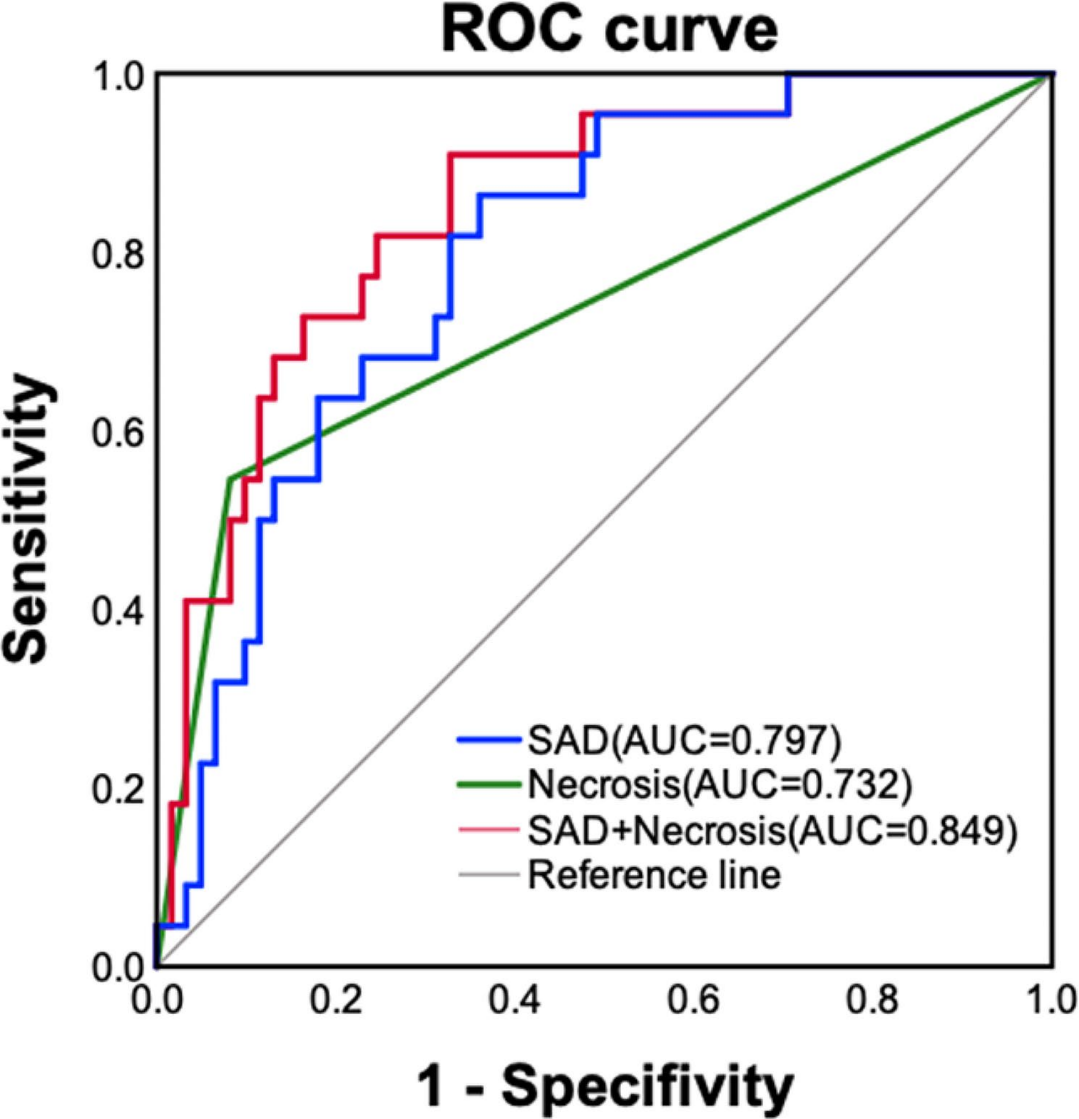
ROC curves of SAD or necrosis only, as well as combined SAD and necrosis for differentiating SUC from CUC

The multinomial logistic regression analysis revealed that only SAD and necrosis emerged as independent predictors for distinguishing between SUC and CUC (Table 3). A tumor with larger SAD was more likely to be SUC rather than CUC (*p* = .014). The optimal threshold value for SAD was 2.39 cm. Consequently, patients with SAD ≥ 2.39 cm exhibited a higher likelihood of being diagnosed with SUC. Additionally, lesions with necrosis were more commonly observed in SUC than CUC (*p* = .003). The ROC curve analysis revealed that this prediction model achieved an AUC of 0.849 (95% CI: 0.754–0.919) with a sensitivity of 67.2% (95% CI: 54 − 78.7%) and a specificity of 90.9% (95% CI: 70.8 − 98.9%), indicating that the model exhibited a robust performance in discriminating between SUC and CUC (Fig. 6).

**Table 3:** Multinomial logistic regression analysis of various radiologic factors

Factors	Multinomial regression			Cutoff	AUC (95%CI)	Accuracy	Sensitivity	Specificity	PPV	NPV
	Category	p	OR (95%CI)							
Location					0.661(0.548–0.761)	0.651	0.639	0.682	0.848	0.405
	Trigone	0.157								
Shape					0.726(0.617–0.818)	0.795	0.869	0.591	0.855	0.619
	Endophytic	0.487								
	Exophytic	0.614								
	Mixed	0.029								
LAD		0.763		3.25	0.788(0.684–0.870)	0.675	0.590	0.909	0.947	0.444
SAD		0.014	1.053(1.011,1.098)	2.39	0.797(0.694–0.877)	0.699	0.639	0.864	0.929	0.463
EVE					0.702(0.591–0.797)	0.711	0.721	0.682	0.863	0.469
	Yes	0.196								
PPS					0.610(0.497–0.715)	0.747	0.902	0.318	0.786	0.539
	Yes	0.204								
Necrosis					0.732(0.623–0.823)	0.819	0.918	0.546	0.849	0.706
	Yes	0.003	7.488(2.001,28.021)							
Hydronephrosis/Ureteral effusion		0.367			0.637(0.524–0.740)	0.723	0.820	0.455	0.807	0.476
Combined model of SAD and Necrosis					0.849(0.754–0.919)	0.735	0.672	0.909	0.954	0.500

Note—LAD, long-axis diameter; SAD, short-axis diameter; EVE, extravesical extension; PPS, pelvic peritoneal spread; OR, odds ratio; 95%CI, 95% confidence interval; AUC, area under the characteristic curve; 95%CI, 95% confidence interval; PPV, positive predictive value; NPV, negative predictive value

## Discussion

It has been reported that the SUC, as a rare subtype of UC, commonly exhibits a more aggressive nature and poorer prognosis, thus necessitating more intensive treatment approaches. However, due to the rarity of SUC, its imaging characteristics, as well as the distinct imaging features for distinguishing SUC from CUC remain unknown. In this study, we compared the clinical and imaging characteristics between bladder SUC and CUC. Clinically, it is well recognized that SUC predominantly affects males and older individuals (median age = 66 years), and is often associated with hematuria [6]. Consistent with the previous report [6], our series included 81.8% male with an average age of 66.0 years, and 81.8% of them presented with hematuria. However, no significant differences regarding of these clinical features were found between the two malignancies. In addition, our findings found that SUC exhibited distinctive imaging characteristics such as a high prevalence in the trigone of the urinary bladder, larger tumor size, irregular shape, obvious invasiveness, low ADC values, VI-RADS ≥ 4, and the presence of necrosis. Furthermore, larger SAD and tumor necrosis on imaging were independent predictors of SUC with an AUC of 0.849 in multinomial logistic regression, indicating its good discriminatory power to distinguish SUC from CUC. Our study contributes to the understanding of the imaging characteristics of SUC and aids in its differential diagnosis from CUC, thus allowing for accurately identifying high-risk patients with SUC and potentially enhancing the precision of treatment and prognosis for patients with SUC [6].

CT and MRI are essential in diagnosis and distinguishing bladder SUC from other conditions. Nevertheless, there is limited literature on the imaging features of SUC, as well as lack of predictive imaging factors for distinguishing bladder SUC from CUC, except a recent review article just mentioned the imaging features of SUC [15], but with few illustrated examples. The article indicated that SUC commonly shows a large tumor with necrosis, invasion of the bladder muscle layer and extravesical tissues [15]. In our study, we analyzed a larger cohort of SUC patients and evaluated a broader range of imaging factors, including tumor location, number, shape, size, margin, enhancement pattern and degree, presence of calcification or hemorrhage or necrosis, DWI and ADC features on MRI, VI-RADS assessment, local tumor invasiveness, and pelvic lymph node metastasis. Although lack of characteristic imaging features, our results demonstrated that bladder SUC tends to locate in the trigone of the urinary bladder with tumorous necrosis and a lower ADC value, commonly exhibit more invasive growth patterns including a larger size, irregular external shape, higher VI-RADS score and local invasion. These relatively distinctive imaging features were consistent with the previous study [15], and aligns with previous studies investigating the images features of the liver, kidney, and pancreatic sarcomatoid carcinomas (SCs) [20–22]. These imaging findings also coincide with the pathologic and clinical feature that bladder SUC usually has heterogenous components mixed with carcinomatous and sarcomatous elements, and it frequently presents with an invasive growth pattern [2, 12].

Our results demonstrated some distinct imaging characteristics such as location, size, shape, ADC value, necrosis, EVE, PPS, and hydronephrosis or ureteral effusion to distinguish SUC from CUC in univariate analysis. Nevertheless, only larger SAD and tumor necrosis in CT or MRI remained as independent predictors for their differential diagnosis with an AUC of 0.849, a sensitivity of 67.2% and specificity of 90.9%. Patients with SUC have larger SAD than that CUC with an optimal threshold of 2.39 cm. Consequently, a bladder lesion with SAD ≥ 2.39 cm exhibited a higher probability of SUC rather than CUC. SAD refers to the perpendicular short axis diameter of a tumor, providing valuable information about the depth and extent of infiltration into the muscular layer and surrounding tissues in bladder tumor. Bladder SUC with larger SAD in our study represents its exophytic or mixed growth pattern, and more invasion into neighboring tissues, which was confirmed by the pathological results that 90.9% (20/22) of SUC in our study demonstrated muscle invasion. Consistent with aforementioned univariate analysis and these pathological results, larger SAD supports the notion that SUC is more likely to exhibit an irregular external shape, aggressive growth, and destructive mass, indicating a tendency to extend beyond the bladder wall compared with CUC [5–7].

In our study, tumor necrosis has emerged as an additional independent imaging factor for discriminating between the two subtypes of bladder cancers. Over half of our patients (54.5%, 12/22) with SUC showed evidence of tumor necrosis on CT or MRI. Bladder tumors exhibiting necrosis tended to be SUC rather than CUC. This result was similar with some previous studies that larger liver, kidney, and pancreatic SCs often exhibit necrosis within the primary tumor [20–22]. A previous study demonstrated that 73% of sarcomatoid renal cell carcinoma exhibited extensive necrosis within the tumor, specifically within the components of sarcomatoid carcinoma [20]. Pathologically, the sarcomatous variant of SUC usually presents as a large, polypoid, and infiltrative lesion with macroscopic evidence of hemorrhage, necrosis, and cavitation [23]. The sarcomatous component of SUC was mainly composed by rapidly proliferating and poorly differentiated cells, and the neovasculature often failed to supply nutrients to support its aggressive growth, thus leading to necrosis [24], which can ultimately contribute to the heightened aggressiveness and poorer prognosis in SUC compared to CUC.

This study has several limitations. Firstly, due to the rarity of bladder SUC, our single-center, retrospective study had a limited number of patients, thereby patient selection bias was inevitable. Future multiple-center investigations with larger cohorts are highly desired to facilitate more robust analyses and to validate our findings. Secondly, some patients in our study lacked the examinations of CT urography and DCE-MRI, which may potentially impact the comprehensiveness of our radiological assessments. CT urography is a useful technique for diagnosis and differential diagnosis of bladder cancer, as well as recognition of anatomic variants [25], and DCE is crucial for diagnosis and VI-RADS scores of bladder cancer [17, 18]. In future, the utilization of advanced imaging modalities, including but not limited to CT urography and DCE-MRI, holds promise in enhancing the diagnostic accuracy of SUC. In addition, future prospective multiple-center trials are expected to explore a more comprehensive understanding and more effective management of this challenging malignancy.

## Conclusion

Bladder SUC exhibits relatively distinct imaging features, including a higher incidence of locating in trigone, a large tumor size, irregular shape, obvious invasiveness, and particularly tumor necrosis. The presence of large SAD and tumor necrosis can serve as independent predictors to differentiate between bladder SUC and CUC. Utilizing these imaging factors, a predictive model has the potential to identify high-risk patients with bladder SUC prior to surgery.

## Data Availability

Not applicable.

## Abbreviations

UC: Urothelial carcinoma
SUC: Sarcomatoid urothelial carcinoma
CUC: Conventional urothelial carcinoma
LAD: Long-axis diameter
SAD: Short-axis diameter
S/L: The ratio of SAD to LAD
EVE: Extravesical extension
PPS: Pelvic peritoneal spread
ADC: Apparent diffusion coefficient
VI-RADS: Vesical Imaging-Reporting and Data System
FSE: Fast spin echo
T2WI: T2-weighted images
T1WI: T1-weighted images
DWI: Diffusion-weighted images
DCE-MRI: Dynamic contrast enhanced MRI
ROC: Receiver operating characteristic
AUC: The area under the ROC curve
